# Ischemia does not provoke the full immune training repertoire in human cardiac fibroblasts

**DOI:** 10.1007/s00210-024-03107-6

**Published:** 2024-04-23

**Authors:** Constantin Mann, Carolin van Alst, Simone Gorressen, Rachel Nega, Dobromir Dobrev, Maria Grandoch, Anke C. Fender

**Affiliations:** 1 Institute of Pharmacology, West German Heart and Vascular Center, Faculty of Medicine, University Duisburg-Essen, Hufelandstr 55, 45147 Essen, Germany; 2 Institute for Pharmacology and CARID Cardiovascular Research Institute Düsseldorf, Medical Faculty and University Hospital Düsseldorf, Heinrich Heine University Düsseldorf, Düsseldorf, Germany; 3 Institute for Translational Pharmacology and CARID Cardiovascular Research Institute Düsseldorf, Medical Faculty and University Hospital Düsseldorf, Heinrich Heine University Düsseldorf, Düsseldorf, Germany

**Keywords:** Trained immunity, Ischemia, Cardiac fibroblasts, Myocardial, Glycolysis, Inflammasome

## Abstract

Trained immunity of monocytes, endothelial, and smooth muscle cells augments the cytokine response to secondary stimuli. Immune training is characterized by stabilization of hypoxia-inducible factor (HIF)-1α, mTOR activation, and aerobic glycolysis. Cardiac fibroblast (CF)-myofibroblast transition upon myocardial ischemia/reperfusion (I/R) features epigenetic and metabolic adaptations reminiscent of trained immunity. We assessed the impact of I/R on characteristics of immune training in human CF and mouse myocardium. I/R was simulated in vitro with transient metabolic inhibition. CF primed with simulated I/R or control buffer were 5 days later re-stimulated with Pam3CSK for 24 h. Mice underwent transient left anterior descending artery occlusion or sham operation with reperfusion for up to 5 days. HIF-regulated metabolic targets and cytokines were assessed by qPCR, immunoblot, and ELISA and glucose consumption, lactate release, and lactate dehydrogenase (LDH) by chromogenic assay. Simulated I/R increased HIF-1α stabilization, mTOR phosphorylation, glucose consumption, lactate production, and transcription of PFKB3 and F2RL3, a HIF-regulated target gene, in human CF. PGK1 and LDH mRNAs were suppressed. Intracellular LDH transiently increased after simulated I/R, and extracellular LDH showed sustained elevation. I/R priming increased abundance of pro-caspase-1, auto-cleaved active caspase-1, and the expression and secretion of interleukin (IL)-1β, but did not augment Pam3CSK-stimulated cytokine transcription or secretion. Myocardial I/R in vivo increased abundance of HIF-1 and the precursor and cleaved forms of caspase-1, caspase-11, and caspase-8, but not of LDH-A or phospho-mTOR. I/R partially reproduces features of immune training in human CF, specifically HIF-1α stabilization, aerobic glycolysis, mTOR phosphorylation, and PFKB3 transcription. I/R does not augment PGK1 or LDH expression or the cytokine response to Pam3CSK. Regulation of PAR4 and inflammasome caspases likely occurs independently of an immune training repertoire.

**Graphical Abstract:**

Ischemia provokes only part of the immune training repertoire in cardiac fibroblasts. Trained immunity in myeloid and non-myeloid cells is triggered by certain infectious and sterile triggers like β-glucan or oxidized LDL, respectively. Key characteristics of immune training are as follows: stabilization of hypoxia-inducible factor (HIF)-1α, mTOR activation, transcriptional induction of lactate dehydrogenase (LDH), phosphoglycerate kinase (PGK)1 and 6-phosphofructo-2-kinase/fructose-2,6-biphosphatase 3 (PFKFB3), increased glycolysis and lactate production, and enhanced cytokine response to a secondary stimulus such as the toll-like receptor agonist Pam3CSK4. Simulated ischemia/reperfusion (SI/R) reproduces some but not all of these features in human cardiac fibroblasts (CF) as indicated with asterisk (*).

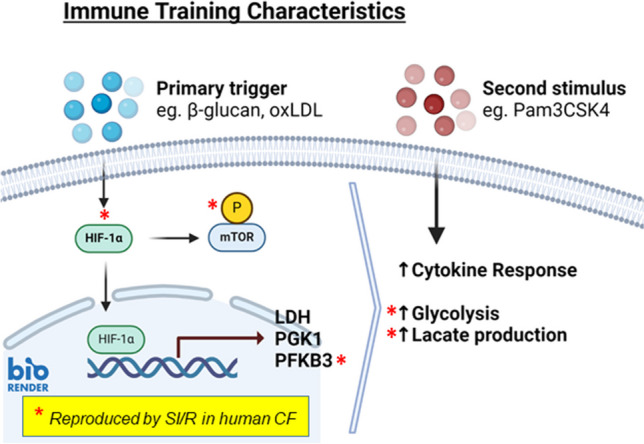

**Supplementary Information:**

The online version contains supplementary material available at 10.1007/s00210-024-03107-6.

## Introduction

The concept of “trained immunity” encompasses a specific constellation of metabolic and epigenetic alterations in myeloid cells that provides an incremental, non-specific protection from secondary infections. Known stimuli of trained immunity in monocytes are β-glucan, a cell-wall constituent of Candida albicans, the bacillus Calmette-Guérin (BCG) vaccine, and sterile triggers including oxidized low-density lipoprotein (oxLDL) (Cheng et al. 2014; Bekkering et al. 2016; Christ et al. 2018; Sohrabi et al. 2018b), catecholamines, and aldosterone (van der Heijden et al. 2020a, 2020b). Monocytes “trained “ with these agents display augmented cytokine responses to secondary, non-related stimuli such as the toll-like receptor (TLR)-2 ligand Pam3CSK4 applied 4–7 days after the first trigger (Sohrabi et al. 2018b; Domínguez-Andrés et al. 2021). Although trained immunity essentially serves to boost host defense, aberrant training can promote the development of a long-lasting hyper-inflammatory and pro-atherogenic phenotype in monocytes and macrophages (Bahrar et al. 2024).

A characteristic feature of trained immunity in monocytes is the switch to aerobic glycolysis, resulting in higher glucose consumption and lactate production, and a reduced NADPH/NADH ratio. Mechanistically, this metabolic remodeling is attributed to stabilization of hypoxia-inducible factor (HIF)-1α and subsequent activation of the AKT/mTOR pathway (Cheng et al. 2014; Sohrabi et al. 2018b; Keating et al. 2020). Immune training is typically verified by increased expression of the HIF-1α-regulated genes encoding lactate dehydrogenase (LDH), phosphoglycerate kinase (PGK)1, and 6-phosphofructo-2-kinase/fructose-2,6-biphosphatase 3 (PFKFB3), reflecting the metabolic switch towards glycolysis (Cheng et al. 2014; Sohrabi et al. 2018a, 2018b; Schnack et al. 2019; Keating et al. 2020).

The capacity to build up an immunological memory was initially attributed to innate immune cells—monocytes, macrophages, and natural killer cells (Netea et al. 2011). Yet the duration of trained immunity was noted to exceed the lifespan of these cells in the circulation, implying that non-immune cells with a longer duration, like stem cells, microglia, and fibroblasts, might possess a similar ability (Hamada et al. 2018, Drummer et al. 2021). The classic immune training protocol has since been established in human aortic endothelial cells (Sohrabi et al. 2020) and human coronary smooth muscle cells (Schnack et al. 2019). Although cardiac fibroblasts (CF) have not been reported to undergo immune training, the fibroblast-myofibroblast transition features epigenetic and metabolic adaptations similar to those associated with the trained immunity phenotype (Lombardi et al. 2019; Hailiwu et al. 2023). Specifically, CF differentiation in the wake of myocardial infraction (MI) involves abnormal aerobic glycolysis supported by the HIF-1α/Akt pathway and the primary target genes Ldha and Pfkb3 (Hailiwu et al. 2023; Wang et al. 2023; Yang et al. 2023).

MI is a condition of acute metabolic stress. Increased glycolysis locally depletes glucose, lactate accumulates, and the elevated proton production causes a fall in pH in the affected myocardium (Stanley 2001; Tian et al. 2023). Experimental MI promotes glucose consumption and upregulation of key glycolytic genes (*Gapdh*, *Ldha*, *Pkm2*) also in monocyte-derived macrophages, thereby regulating their pro-inflammatory M1/M2 phenotypic switch (Gao et al. 2023; Mouton et al. 2023). Accordingly, circulating monocytes in human survivors of acute MI exhibit a sustained pro-inflammatory phenotype (da Silva et al. 2023). Such metabolic reprogramming and augmented inflammatory activation following MI are highly reminiscent of immune training. The high degree of plasticity of CF makes these cells prime candidates to investigate in this regard. The aim of this study was therefore to assess the characteristic features of trained immunity in CF exposed to transient ischemia in vitro and in vivo.

## Methods

### Simulated ischemia/reperfusion (SI/R) in vitro

Human adult primary ventricular cardiac fibroblasts (avHCF, Lot 9602) were purchased from ScienCell Research Laboratories (Provitro AG, Berlin Germany) and maintained in Fibroblast Growth Medium-2 containing 10% fetal bovine serum (FBS, both from ScienCell). Cells were studied at passages 2–3. At approximately 60% confluence, cells were acclimatized to 2% FBS for 2 h and then exposed to a metabolic inhibition buffer that mimics the metabolic conditions of ischemia: proton formation, lactate release, and glucose depletion (Borowski et al. 2010). The simulated ischemia buffer (SIB) contained NaCl 137 mM, KCl 3.5 mM, CaCl_2_ 0.88 mM, MgSO_4_ 0.5 mM, HEPES 4 mM, sodium-l-lactate 20 mM (Santa Cruz Biotechnology, Dallas, USA), 2-deoxy-d-glucose 10 mM (Sigma-Aldrich, St. Louis, MO, USA), FBS 2%, and pH 6.5. The control buffer contained d-glucose 10 mM instead of 2-deoxy-d-glucose, no sodium lactate, and was adjusted to pH 7.4 (Gordon et al. 2003). All compounds were from Merck Millipore (Darmstadt, Germany) or Carl Roth (Karlsruhe, Germany). Buffers were filter-sterilized before use. SIB was previously validated to induce HIF-1α expression in mouse primary CF to the same extent as the direct HIF-inducer deferoxamine (Kleeschulte et al. 2018). After exposure to SIB or control buffers under standard incubation conditions for 4 h (37 °C, 5% CO_2_), CF were gently washed in warm DMEM and returned to normal culture conditions to simulate reperfusion. After 48 h, cells were either harvested or medium was replenished.

### Second-hit stimulation

In alignment with the established protocols of trained immunity in vitro (Sohrabi et al. 2018b, 2020; Schnack et al. 2019; Domínguez-Andrés et al. 2021), CF primed with SIB or control buffer recovered in normal medium for a total of 5 days. They were then either harvested or medium was replenished, additionally supplemented ± Pam3 (TLR2 agonist, 5 µg/mL, BioVision, Waltham, MA, USA) for a further 24 h to elicit a secondary cytokine response. Thereafter, cells and supernatants were collected separately and stored at − 80 °C for analysis.

### Acute MI model in vivo

The acute MI model was essentially applied as previously described and validated (Schneckmann et al. 2023). In brief, male C57BL/6 J mice aged 8–12 weeks (Janvier Labs, Le Genest-Saint-Ile, France) underwent 45 min of left anterior descending (LAD) coronary artery occlusion (closed-chest model) or sham operation and were sacrificed 6 h, 24 h, or 5 days after reperfusion. Mice were excluded from the analysis if weight loss exceeded 20% of body weight, food, and water intake ceased or animals showed no typical voluntary movement. The experiments were performed in line with the principles of the Declaration of Helsinki and were approved by the local authority (Landesamt für Natur, Umwelt und Verbraucherschutz, LANUV Nordrhein-Westfalen, Bezirksregierung Düsseldorf, Az. 81–02.04.2017.A458 and 81–02.04.2022.A297). Animals were sacrificed by cervical dislocation and left ventricles (LV) were rapidly removed, rinsed in PBS, and stored at − 80 °C for analysis.

### Quantitative real-time PCR (qPCR)

Total RNA was extracted from human CF using peqGOLD TriFast™ (Peqlab, Erlangen, Germany) and the GentleMACS Dissociator (Miltenyi Biotech, Bergisch Gladbach, Germany). RNA samples of appropriate purity were reverse transcribed to cDNA using the QuantiTect® Reverse Transcription Kit (Qiagen, Erkrath, Germany) as instructed by the manufacturer. Target mRNA expression was determined using Platinum® SYBR® Green qPCR SuperMix-UDG (Life Technologies, USA, #11,733,038) with ROX reference dye on the StepOnePlus™ Real-Time PCR System (Life Technologies, Singapore, Singapore), using Quantitect Validated Primer Assays from Qiagen. F2rl3, LDH, PGK1, PFKFB3, IL1B, and IL6 mRNA levels were normalized to the three housekeeping genes GATA4, HMBS, and B2M using the ΔΔCt method and meaned.

### Immunoblot

CF pellets were lysed in 1 × Laemmli sample buffer containing 0.01 M Tris, 2% sodium dodecyl sulfate (SDS), and 0.1 M dithiothreitol (DTT), and heated to 95 °C for 5 min. Frozen mouse tissues were crushed under liquid nitrogen and homogenized in Kranias lysis buffer, containing 1.5 M Tris (pH 8.8), 0.5 M EDTA (pH 8.0), 1 M NaF, 20% SDS, 10% glycerol, and 1:10 each of cOmplete™ Mini Protease Inhibitor Cocktail and PhosSTOP™ Phosphatase Inhibitor Cocktail. All chemicals were from Sigma-Aldrich. Homogenates were cleared by centrifugation (15 min, 900 × g at room temperature), supplemented 1:5 with 6 × Laemmli buffer, and heated to 95 °C for 5 min. Western blotting was performed as described (Kleeschulte et al. 2018) using primary antibodies against HIF-1α, mTOR, phospho-(Ser2481)-mTOR, and phospho-(Ser2448)-mTOR (from Cell Signaling Technology Danvers, MA, USA); caspase-1, caspase-4/5/11, caspase-8, and periostin (from Santa Cruz Biotechnology); protease-activated receptor (PAR)4, interleukin (IL)-1β, and IL-6 (from Abcam, Berlin, Germany); lactate dehydrogenase (LDH)-A, alpha-smooth muscle actin (α-SMA), and γ-tubulin (from ThermoFisher Scientific, Waltham, MA, USA). Infrared-coupled secondary antibodies were obtained from LI-COR Biosciences (Bad Homburg, Germany) and diluted 1:1000. Band visualization and quantification were performed using the LI-COR Odyssey platform as described (Fender et al. 2020; Scott et al. 2021); abundance of target proteins was normalized to γ-tubulin or REVERT™ Total Protein Stain (LI-COR). Uncropped immunoblot images are presented in the data supplement.

### ELISA

Conditioned supernatants of human CF cultures were snap-frozen at − 80 °C and assessed by colorimetric/fluorometric assay or ELISA using the following kits as instructed by the manufacturer: Pierce® LDH Cytotoxicity Assay Kit and Human IL-1beta Uncoated ELISA Kit (both from Thermo Fisher), Glucose Assay Kit and Fluorometric Lactate Assay Kit (both from Cell Biolabs, San Diego, CA, USA), Human IL-6 Quantikine ELISA (R&D Systems, Wiesbaden, Germany), Human IL-18 ELISA Kit (MBL International, via Biozol, Eching, Germany).

### Statistical analysis

Data are presented as mean ± standard deviation, where appropriate as fold of the respective controls. Differences between the two groups were determined by the unpaired *t*-test. Statistical testing of more groups utilized one-way analysis of variance (Kruskal–Wallis), with Dunn’s multiple comparison procedure applied as appropriate. * denotes *P* < 0.05.

## Results

### Simulated I/R provokes HIF-1α stabilization, glycolysis, and mTOR activation in human CF

**Fig. 1:**
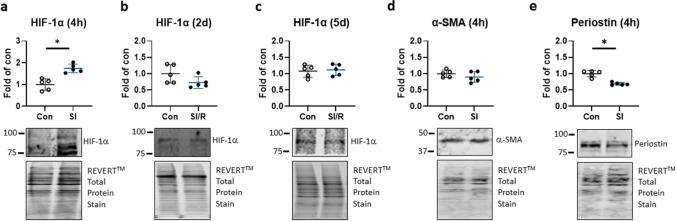
Simulated ischemia/reperfusion (SI/R) stabilizes hypoxia-inducible factor (HIF)-1α in human cardiac fibroblasts (CF). **a** Human CF were subjected to simulated metabolic inhibition (SI) or control (Con) buffers for 4 h and assessed for stabilized HIF-1α by immunoblot **b** directly or after 2 or **c** 5 days of simulated reperfusion (SI/R) in normal culture conditions. **d** Alpha smooth muscle actin (α-SMA) and **e** periostin were determined as myofibroblast differentiation markers in CF exposed to SI or Ctl for 4 h. Proteins of interest were normalized to total protein staining. Data show mean ± SD of *n* = 5 individual experiments, *P < 0.05

**Fig. 2:**
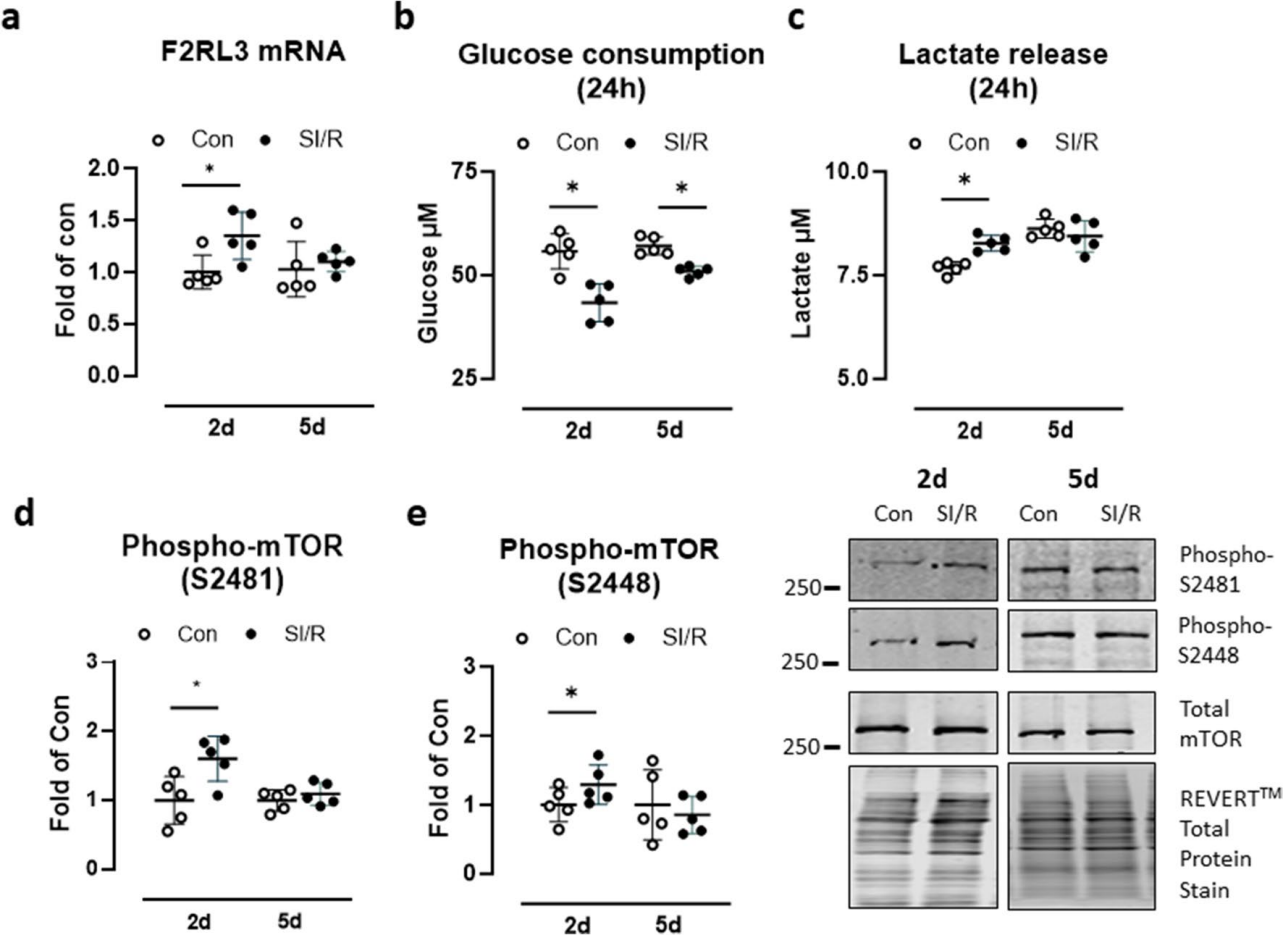
Simulated ischemia/reperfusion (SI/R) promotes glycolysis and mTOR activation. Human cardiac fibroblasts (CF) were exposed to metabolic inhibition or control buffer for 4 h followed by normal culture conditions for up to 5 days. **a** F2RL3 mRNA was assessed on day 2 (d2) and day 5 (5d) of simulated reperfusion. **b** Twenty-four-hour glucose and **c** lactate levels determined in CF supernatants on days 2 and 5 of SI/R versus control. **d** mTOR phosphorylation at serine 2481 and **e** serine 2448 determined by Western blot on days 2 and 5 after SI/R or control treatment, normalized to total mTOR. Data show mean ± SD of *n* = 5 individual experiments, *P < 0.05

Exposure of human CF to the metabolic inhibition buffer that reproduces ischemic conditions (glucose depletion, low pH, extracellular lactate) for 4 h led to a transient stabilization of HIF-1α protein, with restoration of normal HIF-1α protein abundance thereafter (Fig. 1a–c). Cultured CF expressed notable amounts of alpha-smooth muscle actin (α-SMA) and periostin, indicating a degree of myofibroblast differentiation; periostin but not α-SMA showed a lower expression after simulated ischemia (Fig. 1d, e). SI/R was verified to transiently upregulate F2RL3 mRNA, which encodes protease-activated receptor PAR4 and is a regulatory HIF-1α target (Kleeschulte et al. 2018). F2RL3 mRNA was increased on day 2 after SI/R and restored to control levels by day 5 (Fig. 2a). Glucose consumption was significant at days 2 and 5 after SI/R (Fig. 2b); lactate production was also higher in SI/R versus control cells at day 2; by day 5, extracellular lactate levels were comparable (Fig. 2c). The HIF-1α effector kinase mTOR showed increased phosphorylation (phospho/total ratio) at both serine 2481 and serine 2448 on day 2 after SI/R; by day 5, mTOR activation was again comparable to that seen in control CF (Fig. 2d, e).

### Simulated I/R does not induce the full immune training-associated regulatory program in human CF

**Fig. 3:**
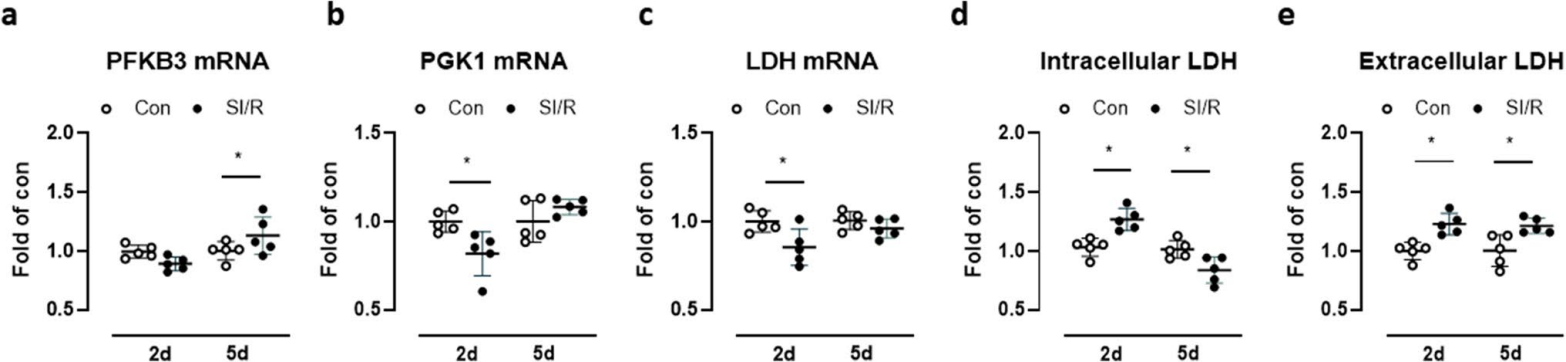
Effect of simulated ischemia/reperfusion (SI/R) on expression of metabolic regulators. Human cardiac fibroblasts (CF) were exposed to metabolic inhibition or control buffer for 4 h followed by normal culture conditions for up to 5 days. **a** PFKFB3, **b** PGK1, and **c** LDH mRNA expression was assessed on day 2 (d2) and day 5 (5d) of simulated reperfusion. **d** LDH levels were determined in CF lysates and **e** supernatants on d2 and d5 after SI/R or control treatment. Data show mean ± SD of *n* = 5 individual experiments, *P < 0.05

The three genes that typify immune training—PFKB3 (6-phosphofructo-2-kinase/fructose-2,6-biphosphatase 3), PGK1 (phosphoglycerate kinase 1), and LDH (lactate dehydrogenase)—were compared in CF challenged with SI/R. PFKB3 mRNA was modestly (non-significantly) lower in SI/R versus control cells on day 2 but upregulated on day 5 (Fig. 3a). Both PGK1 and LDH transcripts were significantly reduced in SI/R cells on day 2 compared to controls and normalized again by day 5 (Fig. 3b, c). Intracellular LDH protein (detected by ELISA) was by contrast higher in SI/R compared to control cells on day 2 but lower on day 5 (Fig. 3d). Extracellular LDH was augmented at both time-points after SI/R compared to control CF (Fig. 3e).

**Fig. 4:**
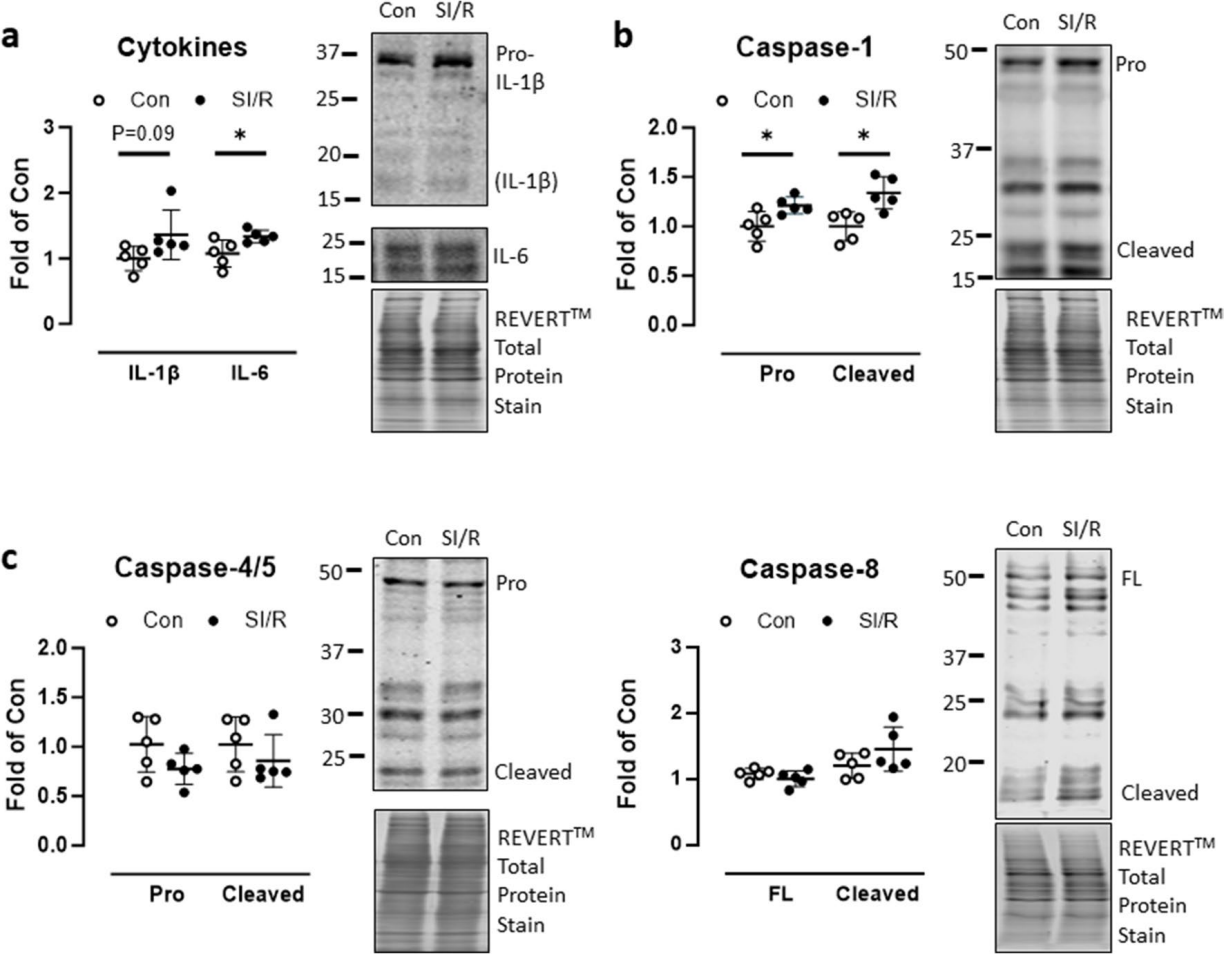
Inflammasome regulation by simulated ischemia/reperfusion (SI/R). Human cardiac fibroblasts (CF) were exposed to metabolic inhibition or control buffer for 4 h followed by normal culture conditions for 5 days. **a** Protein expression of IL-1β and IL-6 was determined on day 5 of simulated reperfusion, normalized to total protein staining. **b** Pro-caspase-1 and auto-activated cleaved caspase-1, **c** pro- and cleaved caspase-4/5, and **d** full-length (FL) and cleaved caspase-8 were determined on day 5 after SI/R or control treatment, normalized to total protein staining. Data show mean ± SD of *n* = 5 individual experiments, *P < 0.05

The canonical (caspase-1) and non-canonical (caspase-4/5/11) inflammasomes have been associated with trained immunity following hepatic I/R injury (Fagenson et al. 2020). In human CF exposed to SI/R, basal expression of IL-1β and IL-6 was modestly increased, significantly so for IL-6 and non-significantly so IL-1β (*P* = 0.09; Fig. 4a). Abundance of both pro-caspase-1 and auto-cleaved active caspase-1 (p20) was higher in SI/R-primed CF compared to controls (Fig. 4b) but for both caspase-4/5 and caspase-8 (alternative inflammasome pathway), expression levels and proteolytic auto-activation were comparable in both groups (Fig. 4c, d).

### Simulated I/R does not augment cytokine responses to secondary Pam3 challenge in human CF

**Fig. 5:**
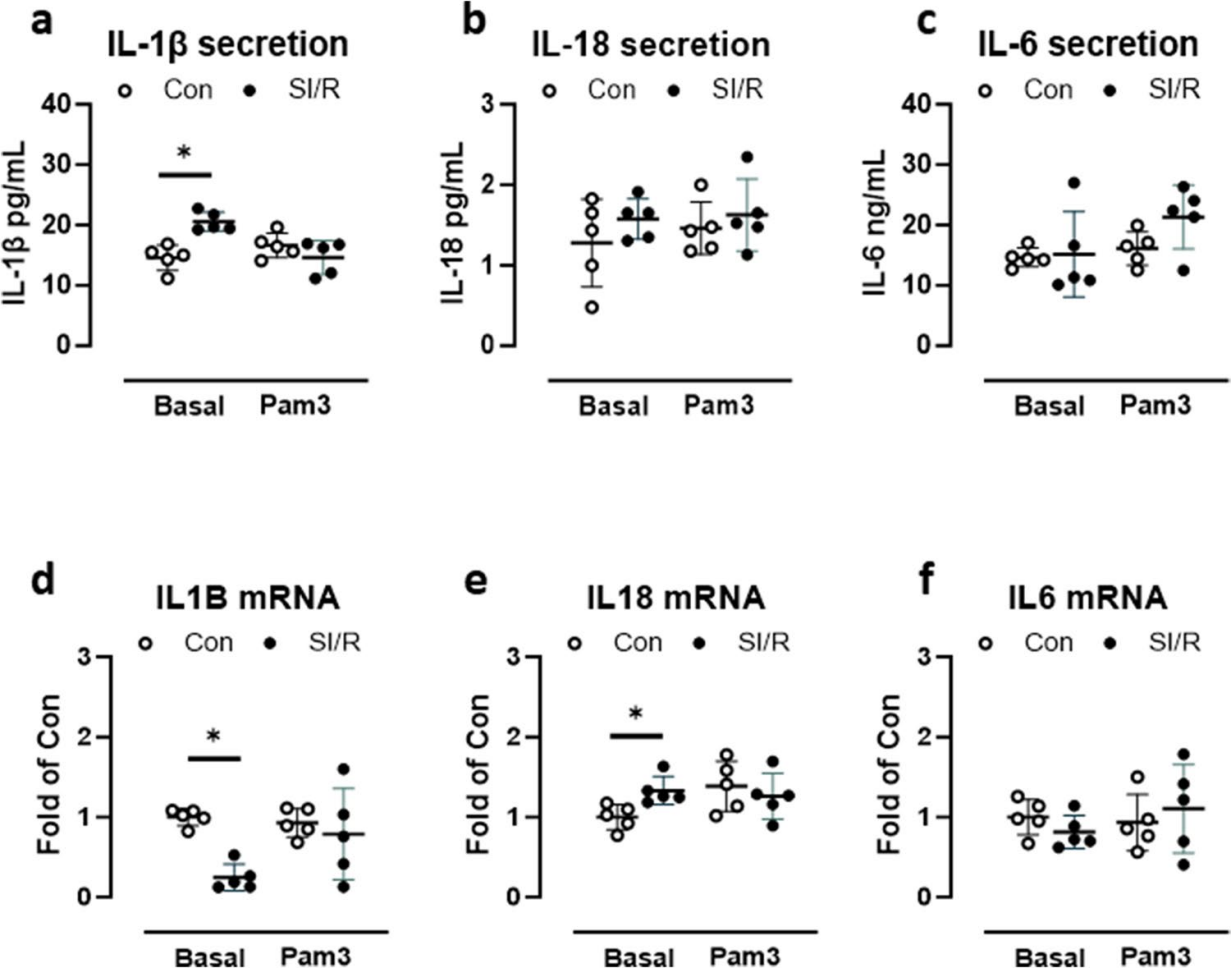
Simulated ischemia/reperfusion (SI/R) does not augment the secondary cytokine response. Human cardiac fibroblasts (CF) were primed with metabolic inhibition or control buffer for 4 h followed by normal culture conditions for 5 days, then re-stimulated with the TLR2 agonist Pam3 or vehicle for 24 h. **a** Basal and Pam3-stimulated levels of IL-1β, **b** IL-18, and **c** IL-6 were determined in CF supernatants; **d** IL1B, **e** IL18, and **f** IL6 mRNA was determined in CF lysates. Data show mean ± SD of *n* = 5 individual experiments, *P < 0.05

CF primed with SI/R displayed significantly higher basal secretion of IL-1β than control cells. Stimulation with the TLR2 agonist Pam3 did not increase IL-1β in either group (Fig. 5a). IL-18 and IL-6 secretion was not significantly modulated by either SI/R priming or by acute exposure to Pam3 (Fig. 5b, c). At the transcript level, basal IL-1β mRNA was suppressed in CF challenged with SI/R compared to control cells; Pam3 had no regulatory effect on IL-1β expression. IL-18 mRNA was by contrast elevated in SI/R-primed cells, but not incrementally increased upon re-stimulation with Pam3 (Fig. 5e). IL-6 transcript was unaffected by either SI/R or acute Pam3 exposure.

### Acute I/R augments myocardial HIF-1α and PAR4 but not LDHA or mTOR in vivo

**Fig. 6:**
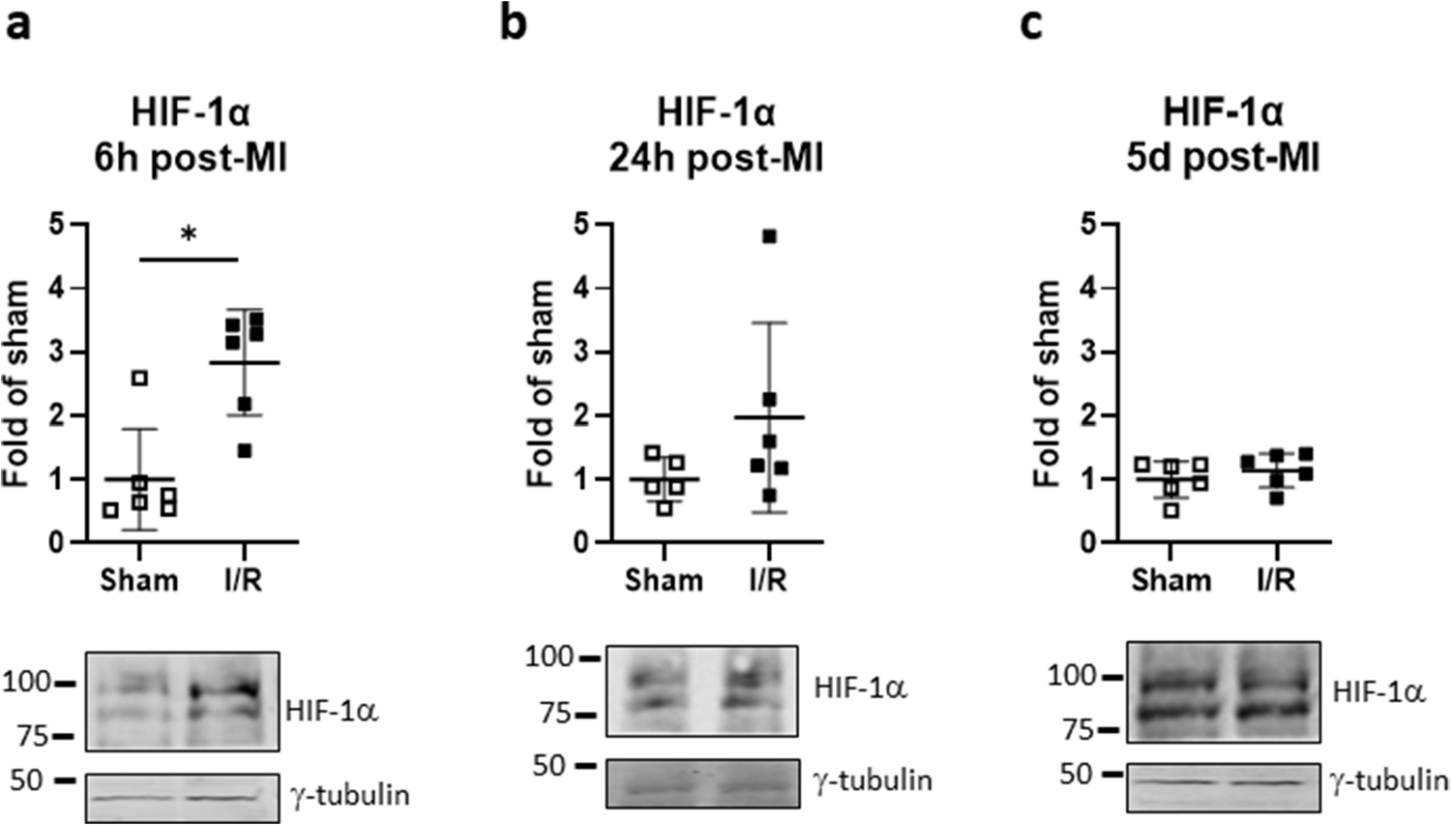
Myocardial ischemia transiently stabilizes hypoxia-inducible factor (HIF)-1α in vivo. Mice were subjected to acute ischemia and reperfusion (I/R) or sham-operated. **a** HIF-1α protein levels, normalized to γ-tubulin, were determined in ventricular lysates after 6 h, **b** 24 h, and **c** 5 days of reperfusion. Data show mean ± SD of *n* = 5–6 individual mice, *P < 0.05

Transient LAD occlusion was validated to increase HIF-1α stabilization in murine myocardium. An approximately threefold increase in HIF-1α abundance was seen after 6 h reperfusion in I/R versus sham hearts, with a decline towards control levels at 24 h and on day 5 after I/R (Fig. 6).

**Fig. 7:**
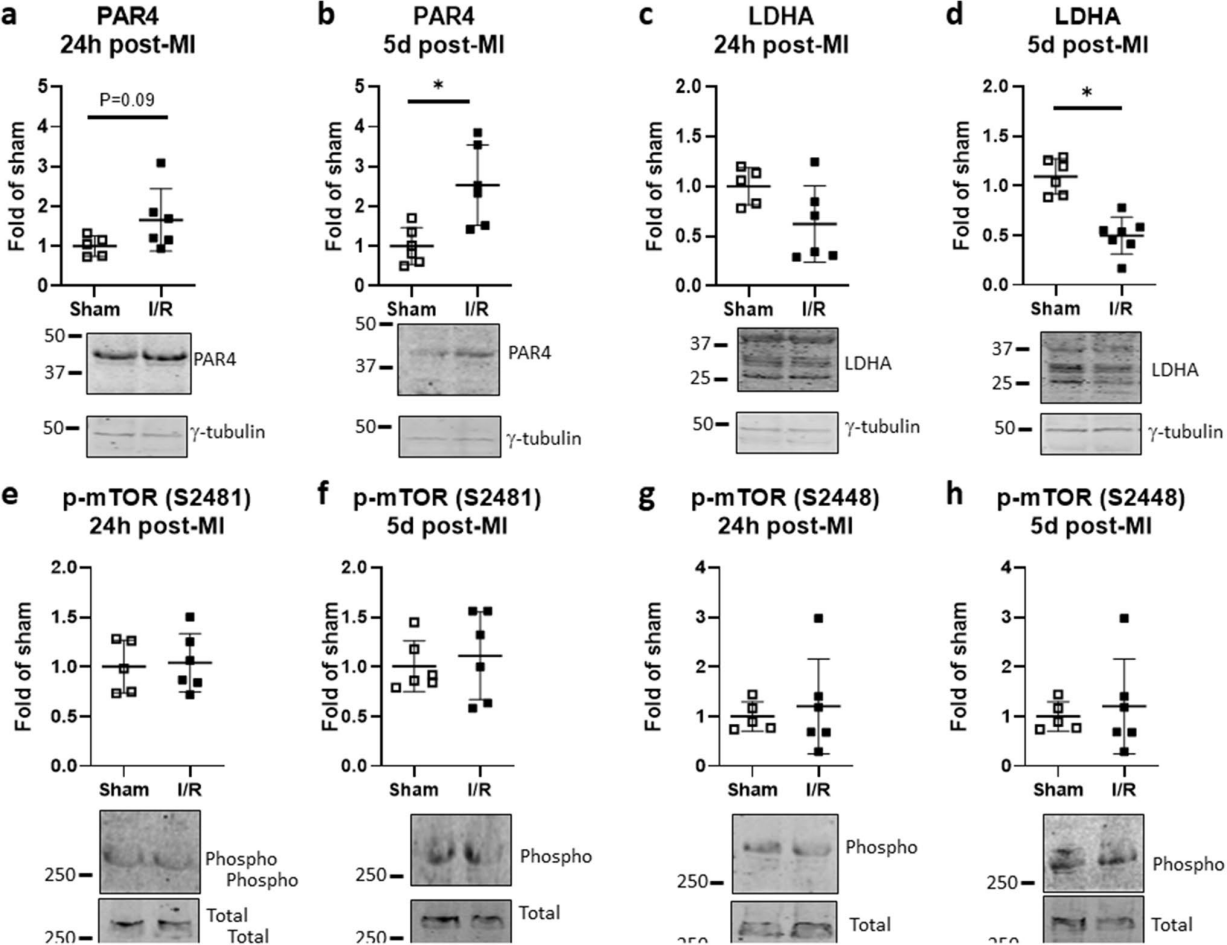
Myocardial ischemia elevates PAR4 but not LDH or phosphorylated mTOR in vivo. Mice were subjected to acute ischemia and reperfusion (I/R) or sham-operated. **a** PAR4 protein expression, normalized to γ-tubulin, was determined in ventricular lysates after 24 h and **b** 5 days of reperfusion. **c** LDHA protein expression, normalized to γ-tubulin, was determined in ventricular lysates after 24 h and **d** 5 days of reperfusion. **e**, **f** mTOR phosphorylation at serine 2481 and **g**, **h** serine 2448, normalized to total mTOR, was determined in ventricular lysates after 24 h and 5 days of reperfusion as indicated. Data show mean ± SD of *n* = 5–6 individual mice, *P < 0.05

PAR4 protein expression increased subsequent to HIF-1α preservation, modestly at 24 h and significantly on day 5 after I/R (Fig. 7a, b). LDHA abundance was by contrast reduced modestly at 24 h and significantly by day 5 (Fig. 7c, d). No difference was seen at either time-point between the groups in terms of mTOR phosphorylation at serine 2481 and serine 2448 (Fig. 7e–h). None of these proteins differed between sham versus I/R after 6 h reperfusion (data not shown).

### Acute I/R promotes multiple inflammasome pathways in mouse myocardium

**Fig. 8:**
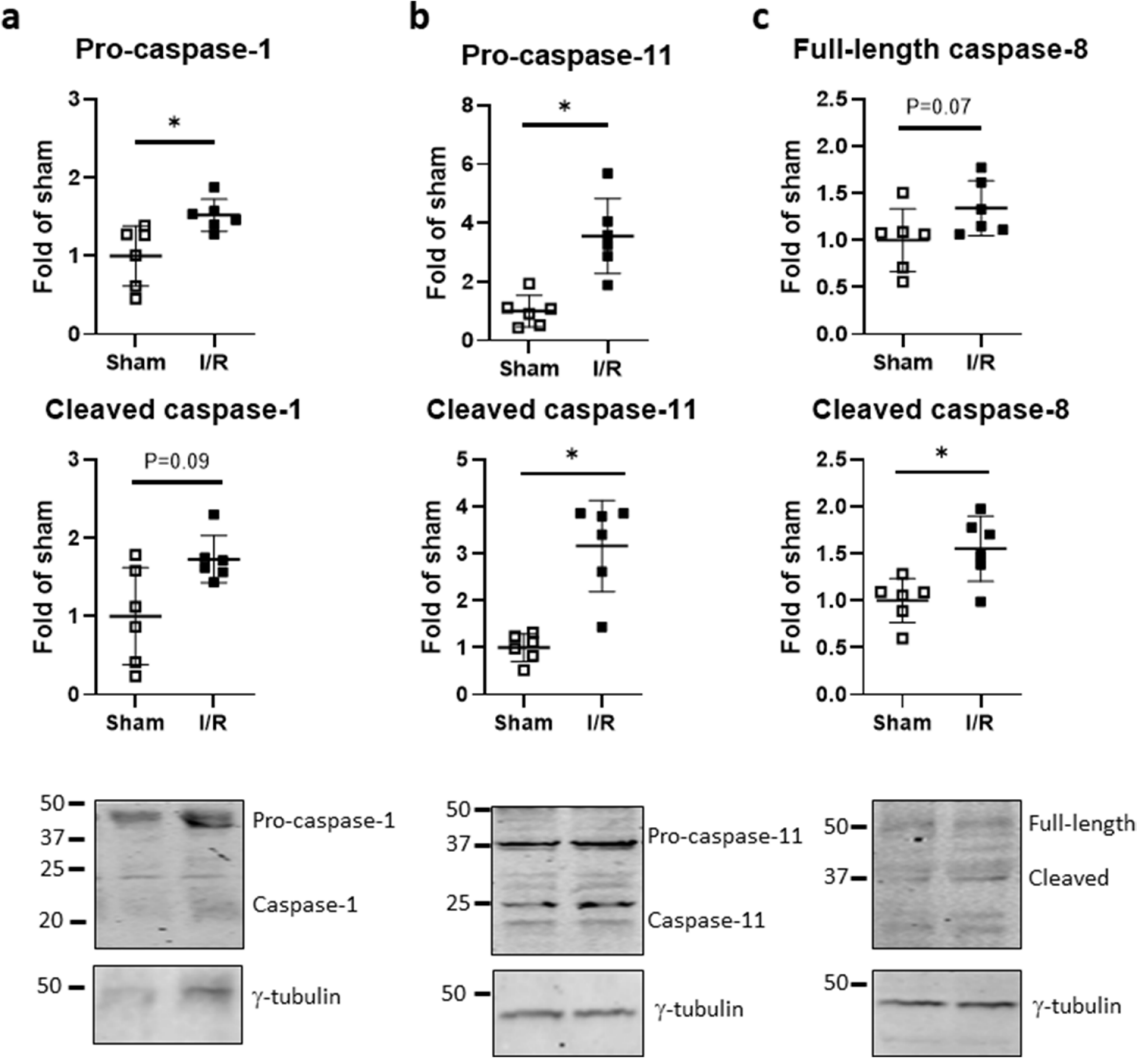
Myocardial ischemia regulates multiple inflammasome effector caspases in vivo. Mice were subjected to acute ischemia and reperfusion (I/R) or sham-operated. **a** Protein levels of precursor and cleaved caspase-1, **b** caspase-11, and **c** caspase-8, normalized to γ-tubulin, were determined in ventricular lysates after 5 days of reperfusion. Data show mean ± SD of *n* = 6 individual mice, *P < 0.05

Acute MI with 5 days of reperfusion increased the abundance of both pro-caspase-1 and proteolytically auto-activated caspase-1 (canonical inflammasome pathway) in mouse myocardium compared to time-matched shams (Fig. 8a). No difference was seen between the groups at either 6 h or 24 h after I/R (data not shown). Precursor and cleaved forms of caspase-11 (non-canonical inflammasome pathway) were also significantly elevated in MI/R versus sham hearts on day 5 (Fig. 8b), with no difference noted before this time-point (data not shown) The alternative inflammasome pathway is represented by caspase-8; full-length caspase-8 was modestly augmented after 5 days of I/R and reperfusion; for cleaved caspase-8, the increase was significant (Fig. 8c). No difference between the groups was observed at the earlier time-points (data not shown).

## Discussion

CF possess a high degree of phenotypic plasticity specifically in the context of MI (Olsen et al. 2017; Cakir et al. 2022). During myofibroblast differentiation, the HIF-1α-activated pathways promote a metabolic shift towards abnormal aerobic glycolysis (Hailiwu et al. 2023; Wang et al. 2023; Yang et al. 2023), reflecting what is seen during immune training of monocytes and certain non-myeloid cells like smooth muscle and endothelial cells. During immune training, an initial challenge with fungal/bacterial triggers or oxidized LDL provokes HIF-1α/mTOR-mediated metabolic and epigenetic reprogramming that culminates in an excessive cytokine response to a subsequent pro-inflammatory stimulus aerobic glycolysis (Sohrabi et al. 2018b, 2020; Schnack et al. 2019). Hindlimb ischemia was recently reported to evoke a trained immunity phenotype in mouse circulating monocytes (Falero-Diaz et al. 2022). We here explored if transient ischemia is also able to evoke the characteristic features of trained immunity in cardiac fibroblasts and myocardium in vivo. A schematic summary of our main findings is depicted in the graphical abstract.

Ischemia/reperfusion was simulated (SI/R) in human CF by 4 h incubation in conditions of metabolic inhibition (glucose deprivation, extracellular lactate, reduced pH) followed by a return to normal culture conditions. The simulated ischemia buffer (SIB) was previously found to upregulate HIF-1α in primary mouse CF to the same extent as the HIF inducer deferoxamine (Kleeschulte et al. 2018). This could now be verified in human CF. HIF-1α protein abundance was approximately doubled after 4 h of SI, with normal levels detected on days 2 and 5 after SI/R. While α-SMA was not regulated in response to 4 h of SI, periostin was suppressed. The significance of this finding is unclear. At least in mice with acute MI in vivo, both periostin and α-SMA were upregulated in cardiac fibroblasts by day 2 (Gil et al. 2022); in mice with cerebral infarction however, periostin levels were transiently suppressed in the ischemic region over 3 h post-insult (Shimamura et al. 2012). Periostin is a secreted extracellular matrix component that contributes to fibrous remodeling in inflammatory settings, so possibly the lower levels observed here are due to acutely accelerated secretion, which we unfortunately did not measure. F2RL3, the gene encoding thrombin receptor PAR4, is a regulatory target of HIF-1α in mouse CF, with increased F2RL3 transcription observed in mouse CF exposed to SIB and in ventricular myocardium of mice with permanent LAD occlusion (Kleeschulte et al. 2018). In this study, human CF upregulated F2rl3 mRNA by day 2 after SI/R, with normalization by day 5.

Glucose consumption was notably accelerated in human CF challenged with SI/R compared to control CF, validating the switch towards increased aerobic glycolysis that characterizes immune training in monocytes and smooth muscle cells. This metabolic adaptation was evident on day 2 and sustained to day 5 after SI/R. Lactate production was also higher on day 2. This is earlier than reported for cells subjected to immune training with oxLDL or β-glucan, where increased extracellular lactate levels were detected 4–5 days after the challenge (Schnack et al. 2019; Sohrabi et al. 2020). In our human CF, we could at this time-point no longer detect any difference between the two groups in terms of extracellular lactate. However, mouse CF driven towards glycolysis by hyperglycemia also does not show augmented extracellular lactate accumulation after several days (Gorski et al. 2019), suggesting that sustained glycolysis may occur without continued lactate extrusion. The prototypical HIF-1α effector kinase mTOR did show increased phosphorylation at both regulatory serines 2481 and 2448 on day 2 after SI/R, and like F2RL3 transcription, was normalized by day 5. Of the classical HIF-regulated target genes that characterize immune training, SI/R was found to induce a delayed increase, evident on day 5, in PFKFB3 mRNA. This is in accordance with PFKFB3 upregulation reported post-MI (Wang et al. 2021) and with the glycolytic switch associated with immune training. By contrast, PGK1 and LDH mRNAs were unexpectedly suppressed; reduced mRNA expression was detected on day 2 after SI/R but restored to control levels by day 5. The divergent regulation of PFKB3 versus PKG1 and LDH contradicts the typical repertoire of features ascribed to immune training. Our data do not clarify if the lower abundance of LDH mRNA on day 2 is due to transcriptional arrest, increased mRNA degradation, or increased translation. We did however observe that intracellular LDH activity increased on day 2, possibly supporting an increased production of protein. Active LDH secreted to the supernatant was also higher in SI/R versus control cells, at both time-points studied. SI/R thus appears to dynamically alter the site and extent of LDH production without overtly affecting lactate levels. Potentially these changes in LDH, especially its release to the extracellular space, are disconnected from an immune training response and rather reflect the typical response of human myocardium to ischemia (Li et al. 2012).

An emerging mechanistic feature of immune training is signaling through the NLRP3 inflammasome, a multimeric platform for auto-proteolytic activation of caspase-1 and subsequent maturation of IL-1β and IL-18. The current evidence for the critical involvement of the NLRP3 inflammasome in trained immunity was recently reviewed (Lee et al. 2023). Canonical NLRP3 inflammasome activation has for example been linked with a trained immunity phenotype arising from a Western diet (WD) in mice (Christ et al. 2018). The authors found that WD provoked a systemic inflammation that reverted when mice were switched back to standard chow, yet myeloid cells retained an exaggerated inflammatory response to innate triggers, which was absent in NLRP3-deficient mice. The implication is that inflammasome activation due to WD primes myeloid cells for secondary immune stimulation, which is highly reminiscent of immune training. In the context of hepatic I/R injury, activation of both canonical (caspase-1) and non-canonical (caspase-4/5/11) inflammasome pathways was associated with upregulation of genes typically associated with immune training, including PGK1 and PFKL (liver-type phosphofructokinase) (Fagenson et al. 2020). In human CF examined on day 5 after SI/R, we found modest increases in the basal intracellular expression of the major inflammasome cytokine product IL-1β and of IL-6. Although small, the increase was statistically significant for IL-6 and nearly so (*P* = 0.09) for IL-1β. In keeping with the increased levels of mature IL-1β, abundance of both pro-caspase-1 and auto-activated caspase-1 were significantly increased in SI/R versus control cells, indicating that the inflammasome was transcriptionally primed and assembled into the multimeric caspase-1-activating platform. Precursor and cleaved forms of caspase-4/5, representing the non-canonical inflammasome pathway, and of caspase-8, representing the so-called alternative inflammasome pathway, did not differ between the groups. Thus, SI/R appears to specifically regulate canonical inflammasome signaling in human CF. Basal secretion of mature IL-1β to the supernatant was also higher in SI/R versus control cells, consistent with augmented caspase-1 auto-activation noted above. However, secondary stimulation of the TLR2 ligand Pam3 did not provoke further IL-1β release either in control cells or in CF primed with SI/R 5 days earlier, indicating that SI/R does not lead to an enhanced cytokine response to Pam3. Production of IL-18 or IL-6 was unaffected by SI/R priming and did not increase incrementally in response to Pam3. Basal IL1B mRNA was curiously reduced in CF previously challenged with SI/R, suggesting that the increased protein levels may arise in part from accelerated translation rather than transcriptional priming. Some degree of transcriptional inflammasome priming may however occur, since SI/R-challenged CF expressed modestly—but significantly—higher levels of IL18 mRNA. A comparably modest, albeit non-significant, increase in IL18 mRNA was also evoked by acute stimulation with Pam3, but this was comparable in both control and SI/R cells. IL6 mRNA was similar in all groups, regardless of SI/R priming or acute secondary stimulation with Pam3. Thus, the exaggerated cytokine response to Pam3 that typifies immune training (Sohrabi et al. 2018b; Domínguez-Andrés et al. 2021) is not reproduced by the ischemic challenge of human CF.

CF are only one of multiple cell types in the heart which could be targeted for immune training upon ischemia. Acute MI was induced in mice by transient LAD occlusion followed by 6 h, 24 h, and 5 days of reperfusion, and representative immune training indices were assessed in ventricular lysates. Compared to time-matched sham-operated mice, mice with acute MI showed transient HIF-1α stabilization, with HIF-1α protein abundance increasing to approximately threefold within 6 h, and declining thereafter. Protein expression of the HIF-1α regulatory target PAR4 was modestly elevated within 24 h of MI, and by day 5, was significantly higher than in the shams. The converse was seen regarding the expression of LDHA. LDHA was recently identified as a critical driver of both immune training in macrophages (Lundahl et al. 2022) and of the metabolic switch to aerobic glycolysis during cardiac fibroblast-myofibroblast transition (Hailiwu et al. 2023). In this study, protein expression of LDHA in mouse myocardium was modestly reduced within 24 h of MI, and significantly so, by around 50%, on day 5 after MI. This contrasts with the typical immune training repertoire. In keeping with this, mTOR phosphorylation at serines 2481 and 2448 was not affected at all post-MI. A strong impact however was seen in terms of inflammasome activation. Precursor and cleaved caspase-1, caspase-11, and caspase-8 were all elevated in mouse myocardium on day 5 after MI compared to shams. Thus, myocardial ischemia provokes both priming and activation of multiple inflammasome pathways, but this occurs uncoupled from other characteristic features of immune training.

A major limitation of our study is the use of isolated CF in 2D culture. The conventional protocols for induction of trained immunity have been applied with comparable outcomes in monocytes in suspension (Sohrabi et al. 2018b) and in adherent 2D cultures (Domínguez-Andrés et al. 2021), as well as in 2D-cultured coronary smooth muscle cells (Schnack et al. 2019) and endothelial cells (Sohrabi et al. 2020). However, cells isolated from their physiological context may not fully reproduce their in situ function and phenotype, and this may explain some of the differences seen between whole hearts and isolated cells in our study. Performing the secondary Pam3 challenge on Langendorf-perfused hearts isolated on day 5 after MI might be a suitable approach to consider in future studies. This would also circumvent the myofibroblast differentiation that CF undergo during isolation, passage, and culture, which basically induces a diseased state per se. In our study, CF expressed notable levels of α-SMA and periostin protein at baseline, indicating at least partial differentiation to myofibroblasts. Since this process itself promotes a metabolic shift in CF (Hailiwu et al. 2023; Wang et al. 2023; Yang et al. 2023), we cannot exclude that part of the SI/R response that we observed was potentiated—or masked—by myofibroblast differentiation driven by the culture conditions. A further limitation to acknowledge is that we are unable to distinguish the contribution of fibroblasts versus other cell types in the whole heart. Future studies might utilize mice with acute MI in vivo and human post-MI cardiac biopsies, both with and without ex vivo Pam3 stimulation followed by single-cell transcriptomics and flow-cytometry assessment. This approach was beyond the scope of our exploratory first-time study on ischemia-evoked immune training.

In conclusion, we find that transient ischemia elicits some of the cellular adaptations that characterize immune training, specifically HIF-1α stabilization and a metabolic switch to aerobic glycolysis with lactate production. In human CF, there is also an increase in mTOR phosphorylation and upregulation of the glycolytic gene PFKB3. However, other features of immune training, such as the transcriptional upregulation of PGK1 and LDH and the augmented cytokine response to secondary Pam3 exposure, are largely lacking. Upregulation of other HIF targets like PAR4, or the activation of multiple inflammasome pathways, appears to occur solely as a response to the ischemic insult per se rather than as part of an immune training repertoire.

## Supplementary Information

Below is the link to the electronic supplementary material.

Supplementary file1 (PDF 1268 KB)

## Data Availability

The data that support the findings of this study are  are available from the corresponding author upon reasonable request.
