# Supplementary material for: Ischemia does not provoke the full immune training repertoire in human cardiac fibroblasts
Source: Naunyn Schmiedebergs Arch Pharmacol. 2024 Apr 23;397(9):7201–12. doi: 10.1007/s00210-024-03107-6 (PMC11422419; doi:10.1007/s00210-024-03107-6)
Supplement: Supplementary file 1 — Supplementary file1 (PDF 1268 KB) [file 210_2024_3107_MOESM1_ESM.pdf]

Ischemia does not provoke the full immune training repertoire in human cardiac fibroblasts

Constantin Mann[1], Carolin van Alst[1], Simone Gorressen[2], Rachel Nega[3], Dobromir Dobrev[1], Maria Grandoch[3], Anke C. Fender[1]

[1]Institute of Pharmacology, West German Heart and Vascular Center, Faculty of Medicine, University Duisburg-Essen, Essen, Germany
[2]Institute for Pharmacology and CARID Cardiovascular Research Institute Düsseldorf, Medical Faculty and University Hospital Düsseldorf, Heinrich Heine University Düsseldorf, Düsseldorf, Germany
[3]Institute for Translational Pharmacology and CARID Cardiovascular Research Institute Düsseldorf, Medical Faculty and University Hospital Düsseldorf, Heinrich Heine University Düsseldorf, Germany

Supplementary figures:

Uncropped immunoblots are depicted, the yellow rectange indicates portion of blot shown in main figures.

Fig. 1

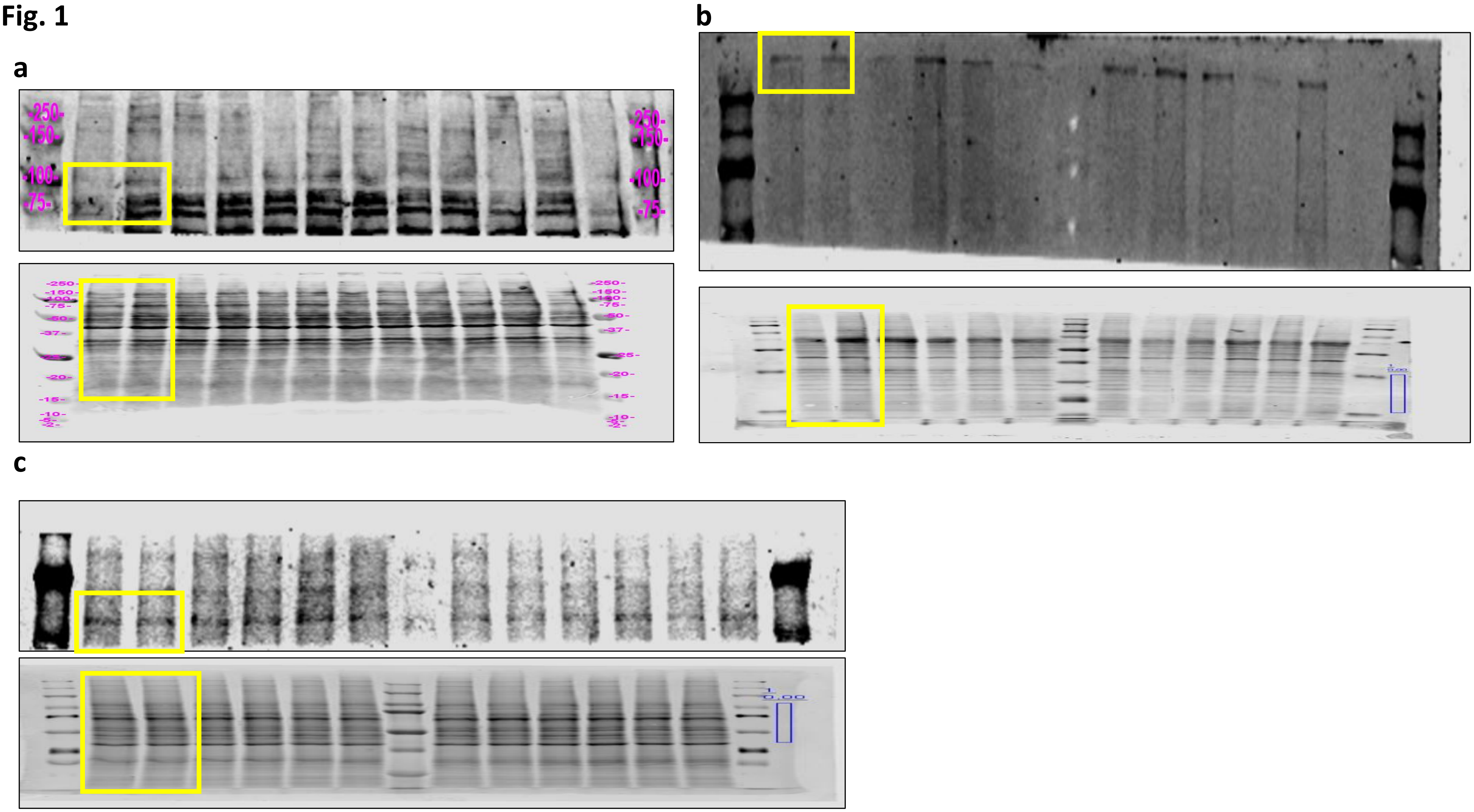

Fig. 1

d

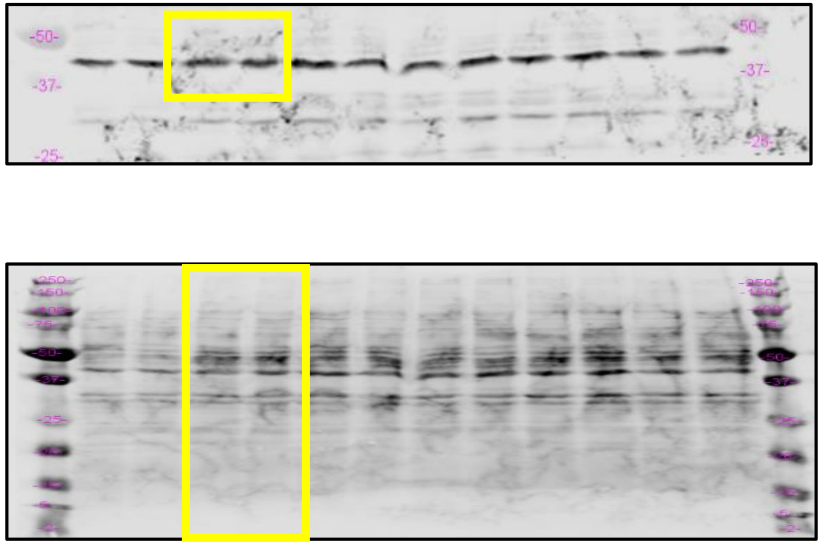

e

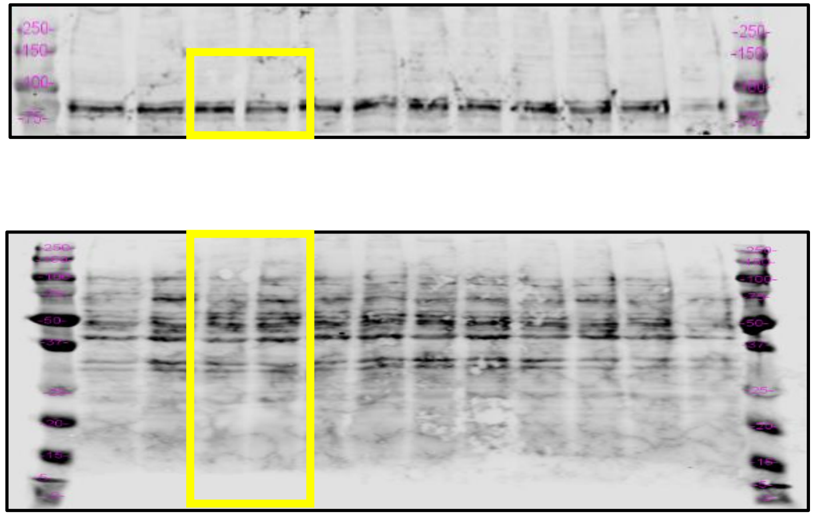

Fig. 2

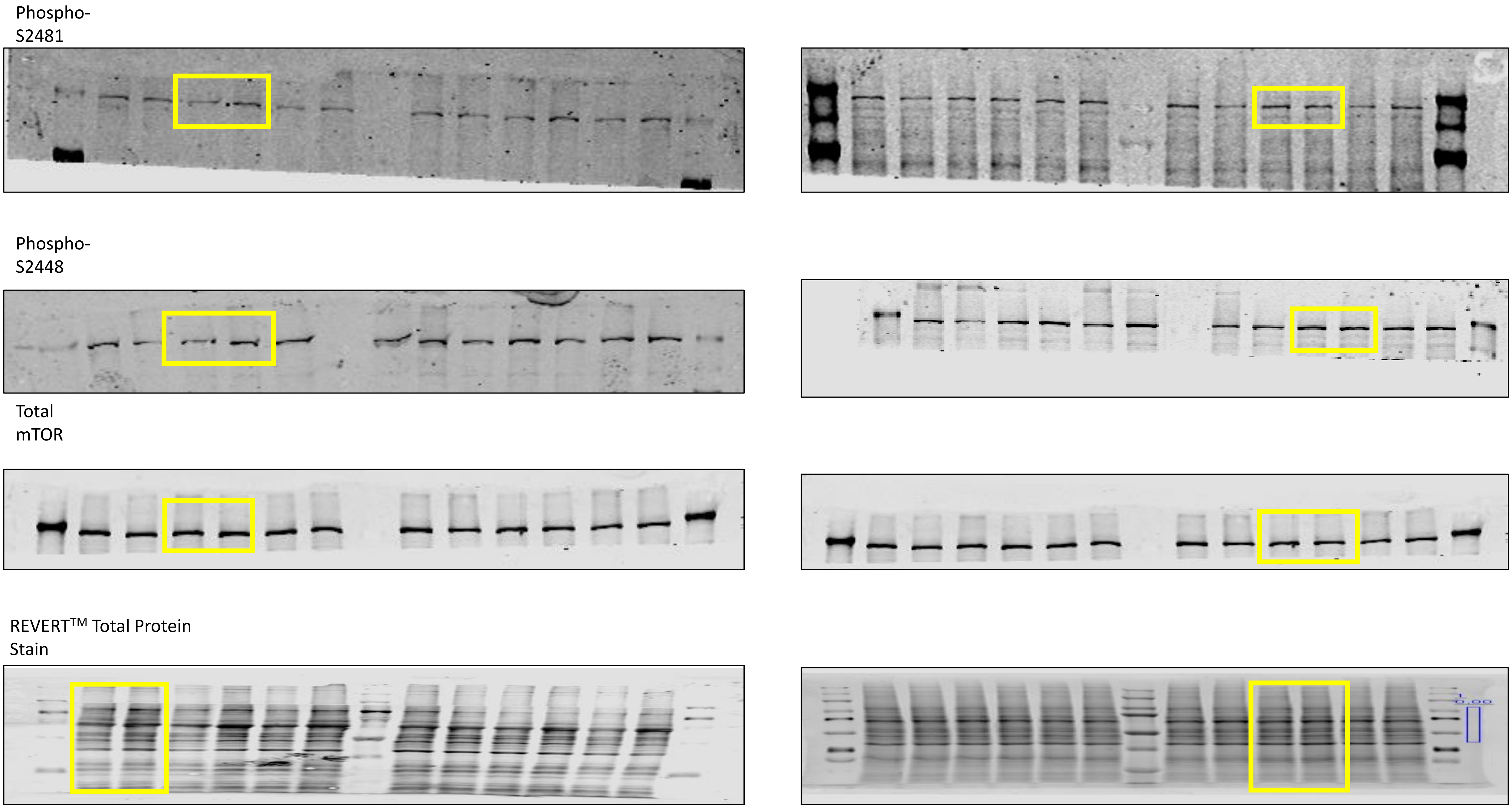
Phospho-
S2481
Phospho-
S2448
Total
mTOR
REVERTTM Total Protein
Stain

Fig. 4

IL-1β

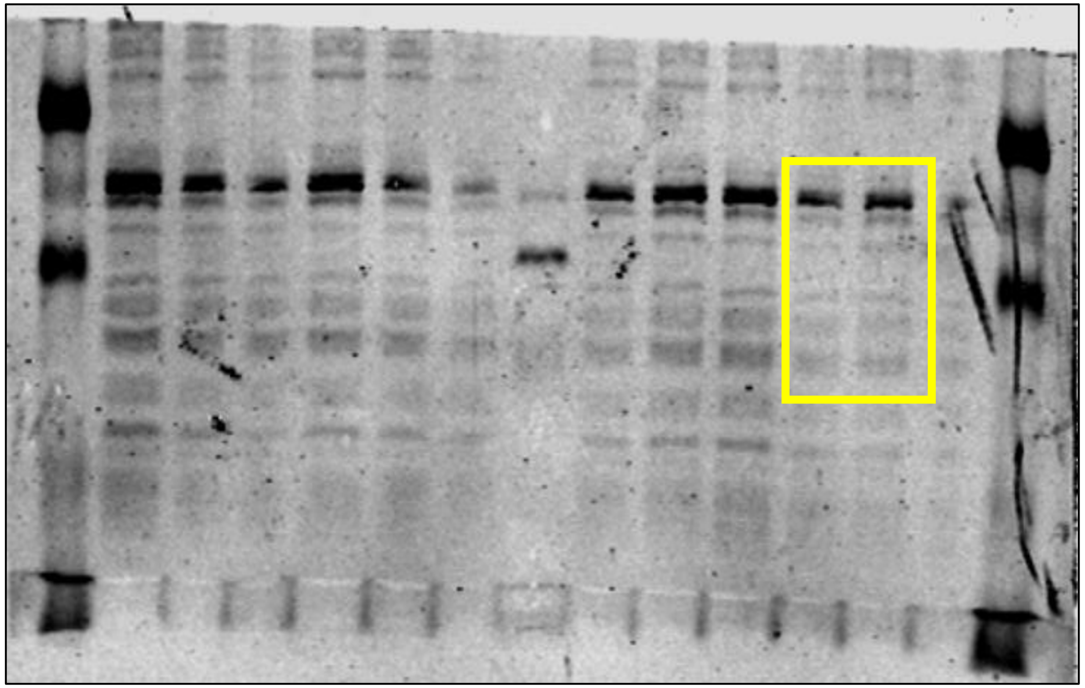

Caspase-1

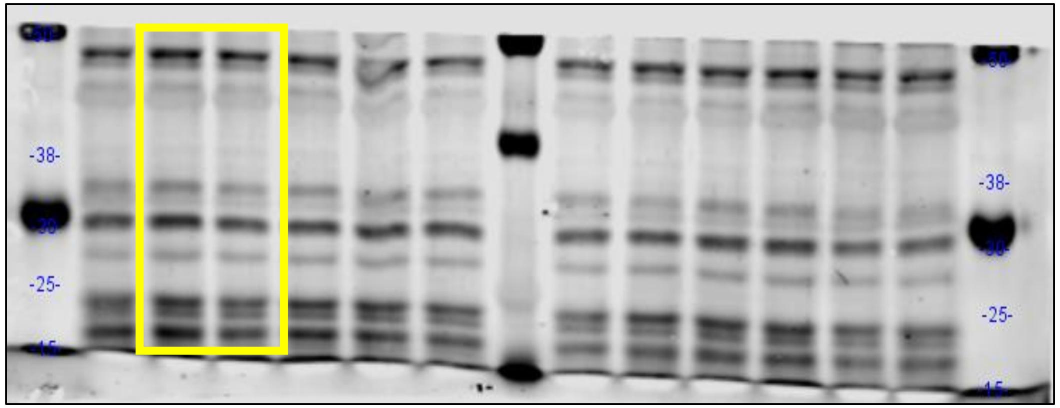

REVERT™ Total Protein Stain

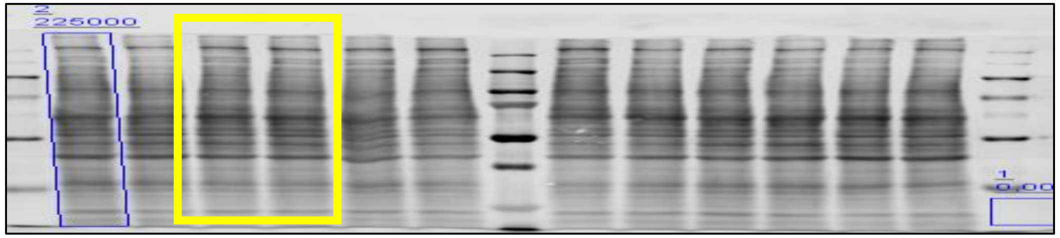

IL-6

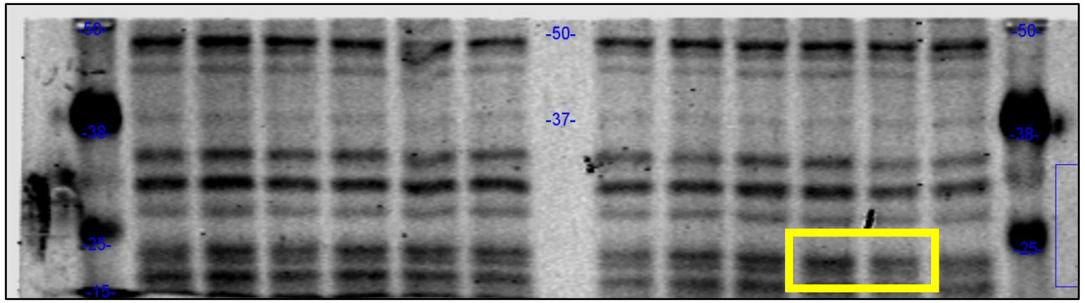

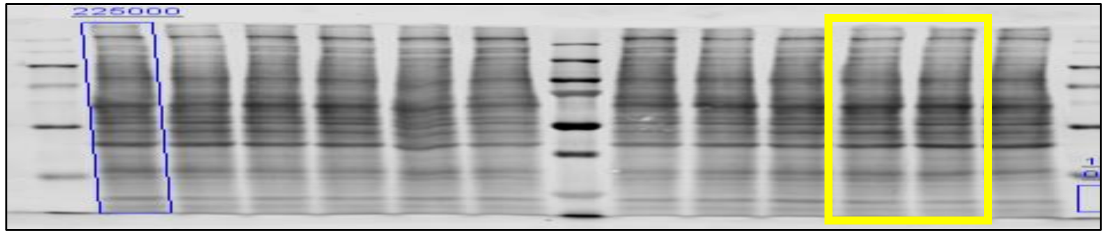

REVERT™ Total Protein Stain

Fig. 6

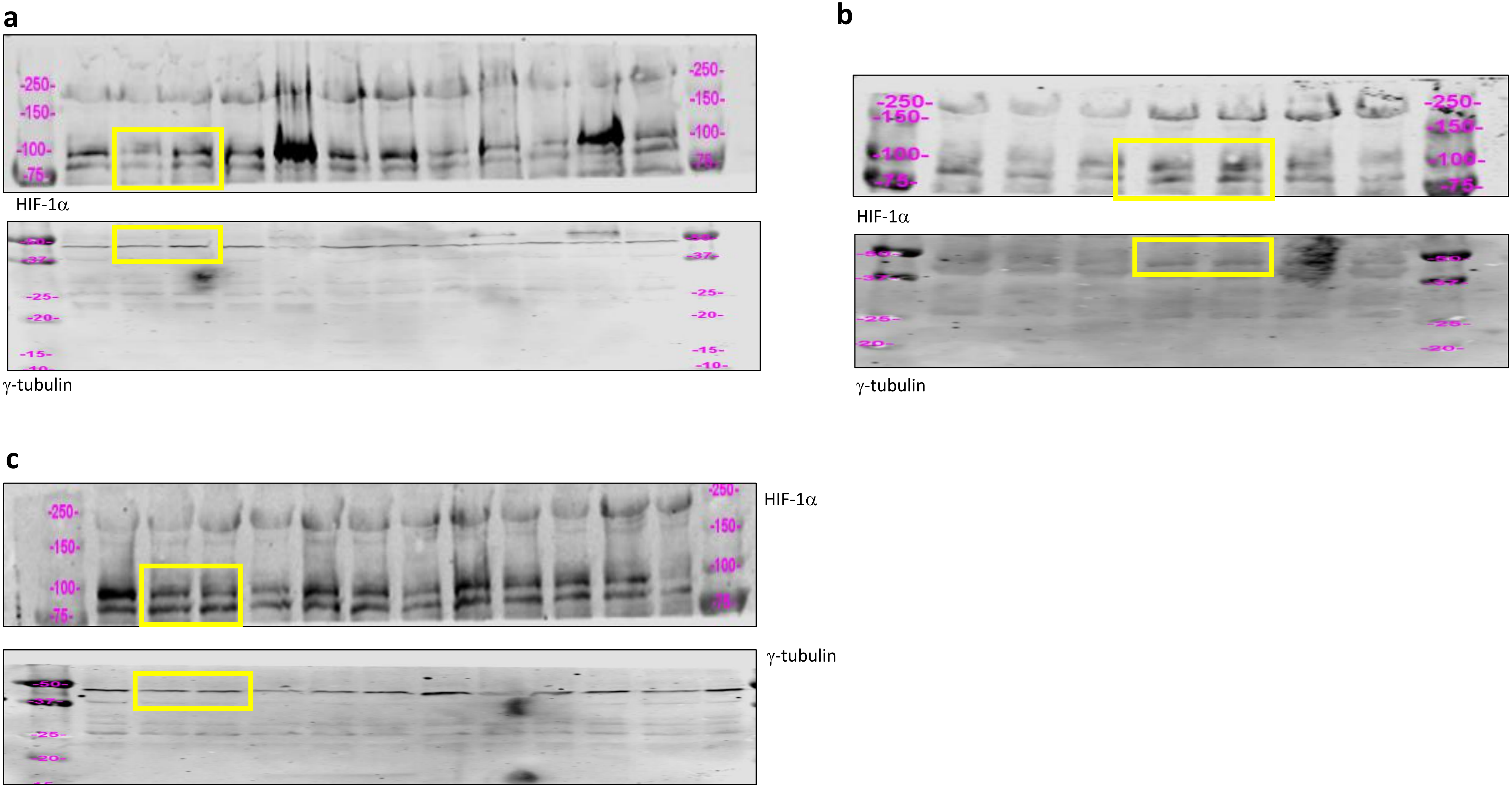

Fig. 7

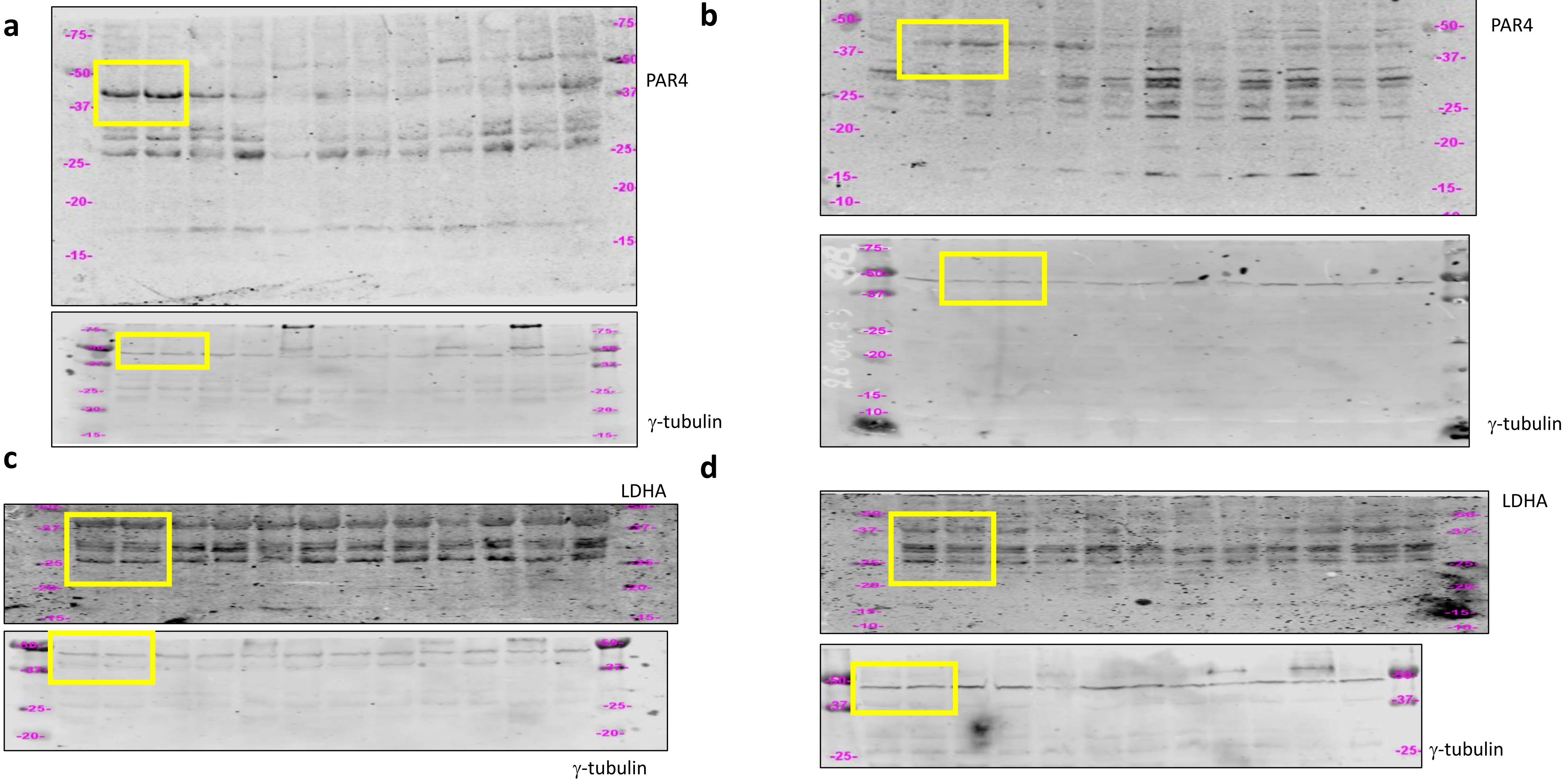
a
PAR4
γ-tubulin
b
PAR4
γ-tubulin
c
LDHA
γ-tubulin
d
LDHA
γ-tubulin

Fig. 7

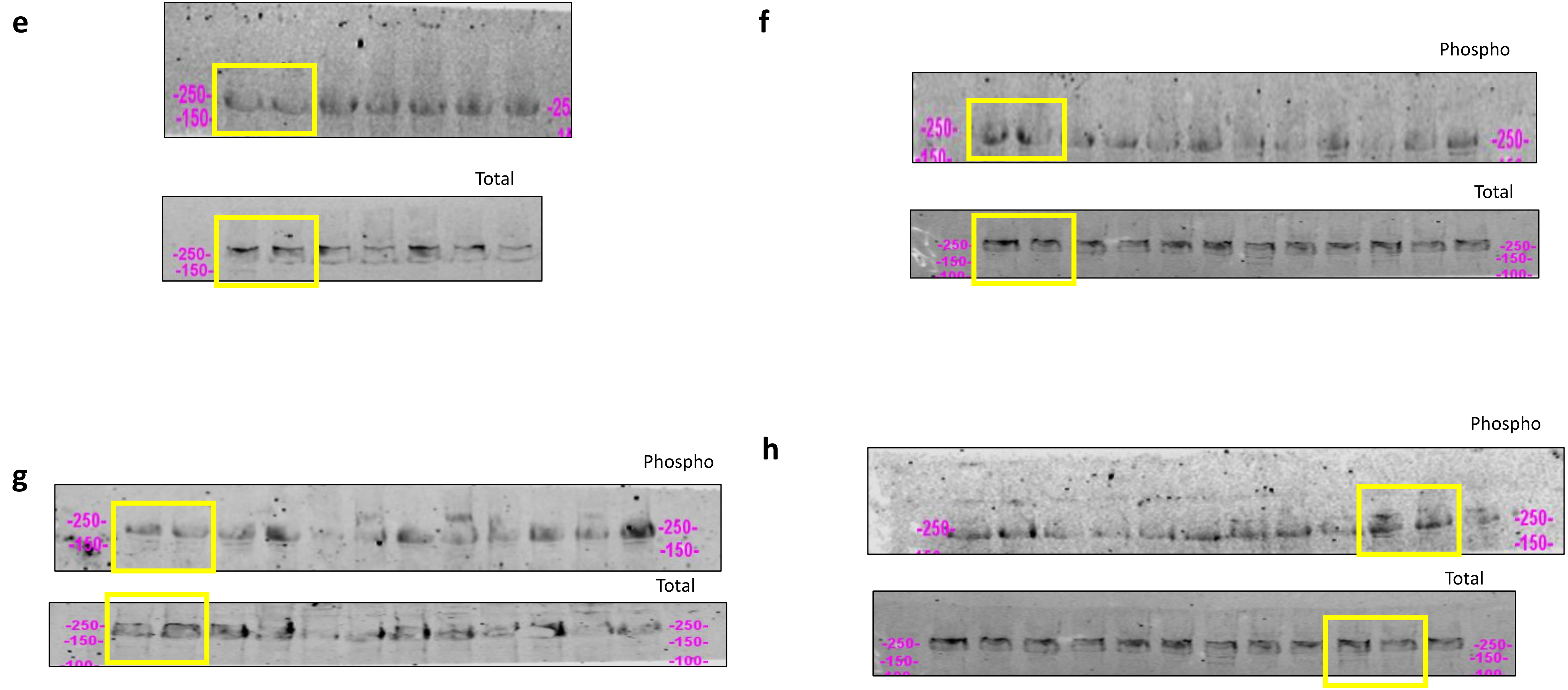
e
Phospho
-250-
-150-
Total
-250-
-150-
f
Phospho
-250-
-150-
Total
-250-
-150-
-100-
g
Phospho
-250-
-150-
Total
-250-
-150-
-100-
h
Phospho
-250-
-150-
Total
-250-
-150-

Fig. 8

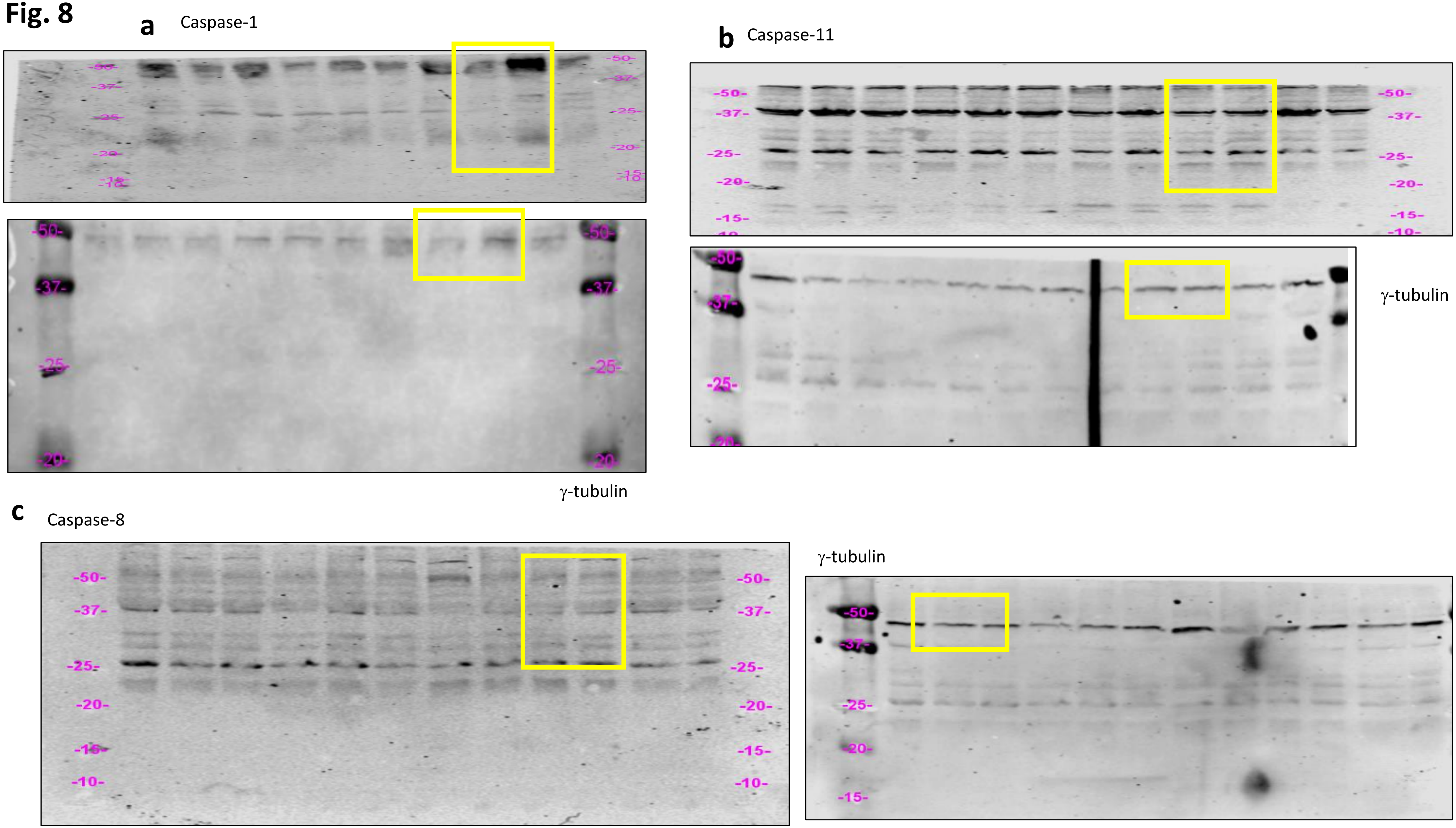
a
Caspase-1
γ-tubulin
b
Caspase-11
γ-tubulin
c
Caspase-8
γ-tubulin
-50-
-37-
-25-
-20-
-15-
-10-
